# Generalized Nonlinear Least Squares Method for the Calibration of Complex Computer Code Using a Gaussian Process Surrogate

**DOI:** 10.3390/e22090985

**Published:** 2020-09-04

**Authors:** Youngsaeng Lee, Jeong-Soo Park

**Affiliations:** 1 Data Science Lab, Korea Electric Power Corporation, Seoul 60732, Korea; yslee82@ejnu.net; 2 Department of Statistics, Chonnam National University, Gwangju 61186, Korea

**Keywords:** best linear unbiased predictor, big data, code tuning, combined data, computer experiments, iteratively re-weighted least squares, Kriging, numerical optimization

## Abstract

The approximated nonlinear least squares (ALS) method has been used for the estimation of unknown parameters in the complex computer code which is very time-consuming to execute. The ALS calibrates or tunes the computer code by minimizing the squared difference between real observations and computer output using a surrogate such as a Gaussian process model. When the differences (residuals) are correlated or heteroscedastic, the ALS may result in a distorted code tuning with a large variance of estimation. Another potential drawback of the ALS is that it does not take into account the uncertainty in the approximation of the computer model by a surrogate. To address these problems, we propose a generalized ALS (GALS) by constructing the covariance matrix of residuals. The inverse of the covariance matrix is multiplied to the residuals, and it is minimized with respect to the tuning parameters. In addition, we consider an iterative version for the GALS, which is called as the max-minG algorithm. In this algorithm, the parameters are re-estimated and updated by the maximum likelihood estimation and the GALS, by using both computer and experimental data repeatedly until convergence. Moreover, the iteratively re-weighted ALS method (IRWALS) was considered for a comparison purpose. Five test functions in different conditions are examined for a comparative analysis of the four methods. Based on the test function study, we find that both the bias and variance of estimates obtained from the proposed methods (the GALS and the max-minG) are smaller than those from the ALS and the IRWALS methods. Especially, the max-minG works better than others including the GALS for the relatively complex test functions. Lastly, an application to a nuclear fusion simulator is illustrated and it is shown that the abnormal pattern of residuals in the ALS can be resolved by the proposed methods.

## 1. Introduction

In many areas of study, modern scientific researchers attempt to develop and use computer code instead of physical experiments when their cost makes them too expensive or infeasible. With the development of computer technology, it is possible to realize the very complex computer simulation which includes a numerical implementation of a physical/mechanistic mode, although it may have some unknown parameters. One of the classic methods for the estimation of unknown parameters in computer code is the nonlinear least squares estimation (NLSE), which minimizes the sum of the squared differences between the experimental observations and the responses of computer simulations. However, if the computer code is complex and time-consuming to execute, the NLSE becomes too expensive or infeasible in terms of time. In these cases, one can use a statistical model to estimate the parameters in the computer code so that the computer simulation model can explain the physical experimental data very well. This procedure is called “calibration” or “code tuning” [1,2,3,4].

The calibration is formally defined as the process of improving the agreement of a code calculation or set of code calculations with respect to a chosen and fixed set of experimental data via the estimation of the parameters implemented in the simulator [5]. Han et al. [1] differentiated between tuning parameter and calibration parameter. In this study, however, the two parameters are treated as the same. We will be lazy by using those two terms (calibration and code tuning) interchangeably.

Cox et al. [3] proposed an approximated NLSE (ALS) for code tuning, in which they employed the Gaussian process (GP) as a metamodel (or a surrogate) of complex computer code. That is, the ALS first fits the GP to the computer data, and then it treats the fitted GP as if it were true simulation code, which makes the ALS computationally feasible. The GP has been successfully used to analyze computer experiments [6,7,8]. In this study, we adopt the same model as a metamodel of computer simulation code.

Our work focuses on calibration in frequentist fashion [9,10,11], rather than a Bayesian one [4,12,13]. Kennedy and O’Hagan [4] introduced a Bayesian calibration in which a bias correction is also done by the GP. Higdon et al. [14] developed a methodology based on a principal component analysis for multivariate computer outputs. Recent contribution to this topic includes the related issues of sequential tuning [15], identifiability [16,17], multi-fidelity [12], efficient designs for calibration [13], large computer experiments [10], information-theoretic approach [18], and surrogate modelling [11,19,20].

In the literature, calibration is often performed within a framework where the code predictions suffer from a systematic discrepancy or bias for any value of parameters. This reflects the view that the mathematical equations underlying the code should not be considered as a perfect model of the real world [4,14]. Even if this framework is more realistic, it is outside the scope of this paper. Thus our presentation is centered on a statistical model which does not include the code bias [3]. However, it would be possible to generalize our framework if the shape of the discrepancy were provided.

The differences between the observations of experiments and the responses of computer simulations are often correlated or do not have equal variance. It is called heteroscedastic problem in a linear model theory [21,22]. In that case, the ordinary least squares approach is not appropriate, so a generalized least squares method is usually employed. In the calibration study, the existing methods including the ALS, however, do not consider the correlation between residuals. Moreover, one potential drawback of the ALS method is that it does not account for uncertainty in the approximation of the computer response by the fitted GP. To address the above two problems, we propose a generalized approximated nonlinear least squares estimation (GALS) method that takes into account the covariance matrix of residuals and the uncertainty due to the approximation.

**Figure 1:**
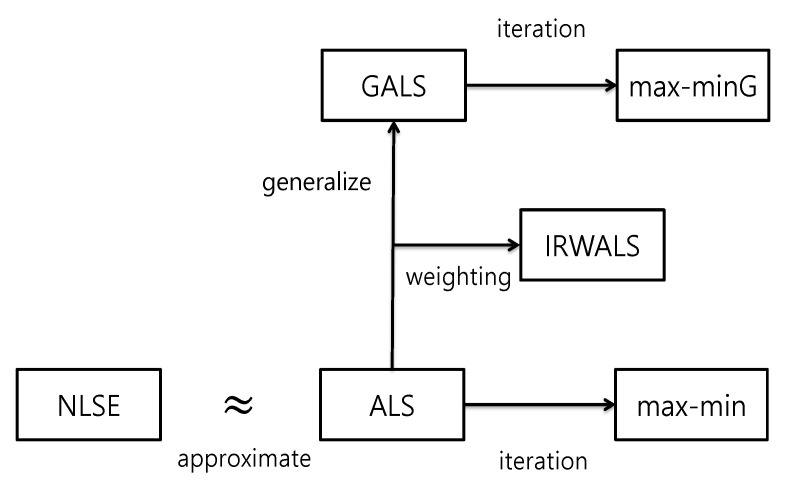
Schematic flow chart related to the proposed methods: Nonlinear least squares estimation (NLSE), approximated nonlinear least squares (ALS), iteratively re-weighted approximated nonlinear least squares (IRWALS), generalized approximated nonlinear least squares (GALS), max-min algorithm, and generalized max-min algorithm (max-minG).

Another potential defect of the ALS is that the surrogate GP is built only once based on the computer data and is not updated thereafter. To solve this difficulty, Seo et al. [2] introduced an iterative version of the ALS. They called it ‘max-min algorithm’. Their method improves the accuracy of calibration and obtains more stable result. In this study, we propose a similar iterative version for GALS method, which is called as ‘max-minG’. In addition, we considered the iteratively reweighted least square method in the ALS setting for calibration of computer code for a comparison purpose. Figure 1 shows the schematic flow chart of our proposed methods. These proposed methods are validated and compared with the existing methods using five test functions, where the true parameters are known a priori.

This paper is organized as follows. Section 2 describes a GP for computer experiments. The ALS approach is introduced in Section 3. The GALS method and its iterative version (the max-minG) are proposed in Section 4 and Section 5, respectively. In Section 6, a test function study is presented. An application to nuclear fusion simulator is given in Section 7. Discussion is given in Section 8, followed by a summary in Section 9. The formula for the covariance matrix of residuals is derived in the Appendix A.

## 2. Gaussian Process Model for Computer Experiments

In this section, we describe the statistical model for the computer simulation data based on the Gaussian process. In many cases, computer simulation code is deterministic. For this reason, Sacks et al. [6] proposed adopting the Gaussian process model (GPM) for computer experiments. The expression of the GP is as follows:

(1) $$Y\left(t\right)=\sum_{i=1}^{p}\beta_{i}f_{i}\left(t\right)+Z\left(t\right),$$

where Y(t) is the response at site t, $\left\{f_{i}\left(\cdot \right)\right\}$ is a set of known functions, β is a vector of unknown regression coefficients, and Z(·) is the Gaussian process with mean zero and covariance $\sigma_{Z}^{2}R\left(t\right)$. Here, the first term represents a linear regression model, and the second term represents the departure from the assumed linear model, which allows us to interpolate between the observed sites. For $t_{i}=\left\{t_{i1},...t_{id}\right\}$ and $t_{j}=\left\{t_{j1},...t_{jd}\right\}$, the covariance between $Z(t_{i})$ and $Z(t_{j})$ is given by

(2) $$\mathrm{Cov}\left(Z\left(t_{i}\right),Z\left(t_{j}\right)\right)=\;V\left(t_{i},t_{j}\right)=\;\sigma_{Z}^{2}R\left(t_{i},t_{j}\right),$$

where $\sigma_{Z}^{2}$ is the process variance of Z(·) and $R(t_{i},t_{j})$ is a correlation function. Our choice is obtained from the Gaussian correlation family denoted by [23]

(3) $$R\left(t_{i},t_{j}\right)=\exp\left(-\sum_{k=1}^{d}\theta_{k}{\left(t_{ik}-t_{jk}\right)}^{2}\right),$$

where $\theta_{k}^{\prime }$s denote non-negative parameters. We define $v^{\prime }\left(t_{0}\right),f^{\prime }\left(t_{0}\right)$ by

(4) $$v^{\prime }\left(t_{0}\right)=\left[V\left(t_{0},t_{1}\right),...,V\left(t_{0},t_{n}\right)\right]$$

and

(5) $$f^{\prime }\left(t_{0}\right)=\left[f_{1}\left(t_{0}\right),...,f_{p}\left(t_{0}\right)\right].$$

Here, $v^{\prime }\left(t_{0}\right)$ is a correlation vector between observed sites and a prediction site $t_{0}$, and $f^{\prime }\left(t_{0}\right)$ is a design vector of $t_{0}$.

If the correlation function R(·,·) is known, the best linear unbiased predictor (BLUP) of $Y(t_{0})$, given observation y, is

(6) $$\hat{Y}\left(t_{0}\right)=\left[v^{\prime }\left(t_{0}\right),f^{\prime }\left(t_{0}\right)\right]{\left[\begin{array}{cc} V & F\\ F^{\prime } & 0 \end{array}\right]}^{-1}\left[\begin{array}{c} y\\ 0 \end{array}\right]=f^{\prime }\left(t_{0}\right)\hat{\beta }+v^{\prime }\left(t_{0}\right)V^{-1}\left(y-F\hat{\beta }\right),$$

where V=V(t,t), *y* is the observations at sites t, $F={\left[f_{j}\left(t_{i}\right)\right]}_{1\leq i\leq n,1\leq j\leq p}$ is an n×p design matrix of observed sites, and $\hat{\beta }={\left(F^{\prime }V^{-1}F\right)}^{-1}F^{\prime }V^{-1}y$ is the generalized least squares estimator of β. *V* is usually unknown and, thus, we estimate the hyper-parameters via the maximum likelihood estimation (MLE) from the data collected at the deisgn sites. Then it are plugged into (6), which makes (6) become the so-called empirical best linear unbiased predictor (EBLUP) of $Y(t_{0})$ [23] or the Kriging in geostatistics. We used the package “DiceKriging” [24] of the R program. In the estimation of the GP parameters, starting values of the numerical optimization are important. In this study, we evaluated the likelihood at 200 random points, and selected the 20 values with the highest likelihood values for starting values.

The prediction model can be determined differently according to a combination of $\beta^{\prime }s$ and $\theta^{\prime }s$ in (1) and (3). In this study, we consider the following model:

(7) $$Y\left(x\right)=\beta_{0}+\beta_{1}t_{1}+...+\beta_{d}t_{d}+Z\left(t\right)+\varepsilon ,\mathrm{with}\;d\;\mathrm{different}\theta^{\prime }s.$$

Of course, other models can be considered, and the models based on variable selection algorithm are also possible [25,26,27]. We refer the readers to References [23,28] for more information on GPM and its application to the design and analysis of computer experiments including calibration.

## 3. Approximated Nonlinear Least Squares

We briefly describe the approximated nonlinear least squares (ALS) method proposed by Reference [3]. The following notations for the computer data are used:

*d*: dimension of input variables t=(c,x) of computer code
*q*: dimension of unknown parameters c
c: unknown parameters to be estimated (*q* dimensional)
$c_{S}$: input variables of computer model corresponding to unknown parameters c (*q* dimensional)
$n_{S}$: number of observations in computer simulations
$x_{S}$: independent variables in computer model (*d*-*q* dimensional)
Y(c,x) or $y_{S}$: computer response for input variables (c,x)

In addition, we use the following notations for the real experimental data:

$n_{E}$: number of observations in real experiments
$x_{E}$: independent variables in real experiments (*d*-*q* dimensional)
$y_{E}$: observations in real experiments

Here, the subscripts *S* and *E* represent the computer simulation and real experiment, respectively. If the computer simulation code explains the real experiment data well, we can approximate $y_{E}$ using the following model:

(8) $$y_{E}=Y\left(c,x_{E}\right)+e,$$

where *e* is assumed to be independent and identically distributed $N(0,\sigma_{E}^{2}I)$, and $\sigma_{E}^{2}$ is the variance of real experiments.

When the computer code is very time-consuming to execute, it is almost impossible in terms of time to optimize some quantity directly from the code. For this case, the ALS uses a GPM as a statistical metamodel or a surrogate of computer code. The ALS first fits the GPM for the computer data by estimating the GP parameters using the MLE. Then, it treats the fitted model as if it were true computer code. The ALS finds $\hat{c}$ that minimizes the following approximated residual sum of squares (ARSS);

(9) $$ARSS\left(c\right)=\sum_{i=1}^{n_{E}}{\left[y_{E,i}-\hat{Y}\left(c,x_{E,i}\right)\right]}^{2},$$

where $\hat{Y}\left(c,x_{E}\right)$ is the EBLUP of $Y(x_{0})$, as in Equation (6).

The advantage of this method is that it does not require any additional execution of the computer code to evaluate ARSS(c) after the prediction model is built from a computer data set. Because no explicit solution is available to minimize ARSS(**c**), we use the quasi-Newton method in “optim” package of R program [29].

Sometimes, the residuals $y_{E}-\hat{Y}\left(c,x_{E}\right)$ seem to be correlated or do not have constant variance. The ALS, however, do not take into account the correlation between residuals for calibration. In that case, an approach dealing with the correlation should be more appropriate. Moreover, a potential drawback of the ALS is that it does not account for uncertainty in the approximation of $Y_{C}$ by $\hat{Y}$. We expect this drawback can be handled by the method presented in the next section in which the covariance matrix of the residuals $y_{E}-\hat{Y}\left(c,x_{E}\right)$ is considered. An improvement point of the ALS is that the surrogate $\hat{Y}$ is built only once and is not updated thereafter. This can be addressed by the iterative version of the proposed method.

## 4. A Generalized ALS

Here, we propose a new method, the generalized approximated nonlinear least squares estimation (GALS) by considering the covariance matrix of residuals. The GALS method finds $\hat{c}$ which minimizes the following generalized ARSS(**c**):

(10) $$GARSS\left(c\right)={\left[y_{E}-\hat{Y}\left(T_{E}\right)\right]}^{\prime }K^{-1}\left[y_{E}-\hat{Y}\left(T_{E}\right)\right],$$

where $T_{E}={\left\{t_{E,1},t_{E,2},...,t_{E,n_{E}}\right\}}^{\prime }$ is an $n_{E}\times d$ matrix for $t_{E,i}^{\prime }=\left(c,x_{E,i}\right)$, and $n_{E}\times n_{E}$ matrix *K* is the covariance matrix of residual $y_{E}-\hat{Y}\left(T_{E}\right)$ given by

(11) $$K=E\left[\left(y_{E}-\hat{Y}\left(T_{E}\right)\right){\left(y_{E}-\hat{Y}\left(T_{E}\right)\right)}^{\prime }\right].$$

Through some calculations presented in the Appendix A, *K* is written as follows:

(12) $$K=\sigma_{Z}^{2}\left(\sigma_{Z}^{-2}\Sigma \left(T_{E}\right)+\gamma_{E}I\right),$$

where

(13) $$\sigma_{Z}^{-2}\Sigma \left(T_{E}\right)=R\left(T_{E},T_{E}\right)-\left(U,F_{E}\right){\left[\begin{array}{cc} V_{S} & F_{S}\\ F_{S}^{\prime } & 0 \end{array}\right]}^{-1}\left(\begin{array}{c} U^{\prime }\\ F_{E}^{\prime } \end{array}\right),$$

where $\gamma_{E}=\sigma_{E}^{2}/\sigma_{Z}^{2}$, $F_{S}$ is an $n_{S}\times p$ design matrix of computer simulations, and $F_{E}$ is an $n_{E}\times p$ design matrix for real experiments, respectively. See the Appendix A for derivation of the above form. Here, $U=\left[V\left(T_{E,i},T_{S,j}\right)\right],i=1,...,n_{E},j=1,...n_{S}$ is the $n_{E}\times n_{S}$ correlation matrix between the observations of real experiments and the responses of computer simulations, and $V_{S}=\left[V\left(T_{S,i},T_{S,j}\right)\right],i=1,...,n_{S},j=1,...n_{S}$ is the $n_{S}\times n_{S}$ correlation matrix between the responses of the computer simulations, where $T_{S}={\left\{t_{S,1},t_{S,2},...,t_{S,n_{S}}\right\}}^{\prime }$ for $t_{S,i}^{\prime }=\left(c_{S},x_{S,i}\right),i=1,...n_{S}$. It is notable that the similar matrix was considered by Reference [30] in Bayesian framework for diagnostics of the GPM, not for the code tuning.

For the calculation of Equations (12) and (13), the quantities $R\left(T_{E},T_{E}\right),U,V_{S},\sigma_{Z}^{2}$, and $\gamma_{E}$ have to be specified. In this study, the parameters $\theta^{\prime }s$ and ${\hat{\gamma }}_{S}^{2}$ are inserted by the MLE calculated from the computer data using Equation (7), while ${\hat{\gamma }}_{E}$ is estimated from real data using the simple model, as follows:

(14) $$Y\left(x\right)=\beta_{0}+Z\left(x\right),\mathrm{with}\;d\;\mathrm{different}\theta^{\prime }s.$$

## 5. Iterative Algorithm For GALS

In this section, we consider the iterative version of GALS. Seo et al. [2] proposed so-called the max-min algorithm which is an iterative version of the ALS. In the max-min algorithm, the tuning parameters and GP parameters are alternatively re-estimated and updated by maximum likelihood estimation and the ALS method. This algorithm uses both computer and experimental data repeatedly until convergence. This algorithm is motivated by the iteratively reweighted least squares (IRLS) method in regression analysis. Seo et al. [2] showed that the max-min performs better in calibrating as well as in surrogating the computer code than the ALS. We present a version of the max-min algorithm applied to the GALS, which is referred to the ‘max-minG’. The steps are given in the following;

**Step 1**: Estimate the GP parameters ${\hat{\beta }}^{\prime }s$, ${\hat{\theta }}^{\prime }s$, and ${\hat{\sigma }}_{Z}^{2}$ in Equation (2) and (7) using MLE for the computer simulation data ($T_{S}=\left(c_{S},x_{S}\right)$ and $y_{S}$) only.
**Step 2**: Finds $\hat{c}$, which minimizes the GARSS(**c**) in Equation (10) with the estimates ${\hat{\beta }}^{\prime }s$, ${\hat{\theta }}^{\prime }s$, and ${\hat{\sigma }}_{Z}^{2}$ from Step 1.
**Step 3**: Estimate the GP parameters ${\hat{\beta }}^{\prime }s$, ${\hat{\theta }}^{\prime }s$, and ${\hat{\sigma }}_{Z}^{2}$ for the combined data ($T_{B}$ and $y_{B}$), where $T_{B}=\left(\begin{array}{c} T_{E}^{*}\\ T_{S} \end{array}\right),y_{B}=\left(\begin{array}{c} y_{E}\\ y_{S} \end{array}\right),T_{E}^{*}={\left\{t_{E,1}^{*},...,t_{E,n_{E}}^{*}\right\}}^{\prime }$, and ${t_{E,i}^{*}}^{\prime }=\left(\hat{c},x_{E,i}\right)$. Here, $\hat{c}$ is the estimates obtained from previous step.
**Step 4**: Finds $\hat{c}$ which minimizes the GARSS(**c**) in Equation (10) with the estimates ${\hat{\beta }}^{\prime }s$, ${\hat{\theta }}^{\prime }s$, and ${\hat{\sigma }}_{Z}^{2}$ from Step 3.
**Step 5**: Repeat Steps 3 and 4 until $\;\sum_{i=1}^{q}|{\hat{c}}_{i}^{old}-{\hat{c}}_{i}^{new}\left|\;/\;\right|{\hat{c}}_{i}^{old}|\;<\epsilon$.

In Step 1 and 3, the prediction model is built by the GP model as in Equation (7) with β’s and *d* different θ’s. Steps 2 and 4 are the same in minimizing GARSS(c), but in the prediction $\hat{Y}\left(x_{E}\right)$, Step 2 uses only computer data while Step 4 uses the combined data. Steps 1 and 3 are the same in obtaining the MLE by maximizing the likelihood function, but Step 1 uses only computer data while Step 3 uses the combined data. The likelihood function in Step 3 is $L(\beta ,\;\theta \;;\;\hat{c},\;y_{B},\;x_{B})$. The subscript *B* stands for the combined (or both) data. For the optimizations in Steps 2, 3 and 4, quasi-Newton numerical algorithms were employed.

In each iteration of Steps 3 and 4, the combined data and the parameters in a metamodel are updated, so we expect it to positively affect the estimation of tuning parameter c. Our expectation comes from the fact that the iterative algorithm uses the combined (computer and real experimental) data in building a metamodel while the ALS and the GALS use the computer data only. Adding relevant data usually improves the prediction ability of the metamodel. The advantage of the max-minG is that both of computer data and real experiment data are used for building a metamodel. Thus, the uncertainty in the approximation Y using the metamodel gets smaller than those of the ALS and the GALS, because the ALS or the GALS builds a surrogate using computer data only [2]. We expect the max-minG method works well in calibrating as well as in surrogating the computer code.

## 6. Test Function Study

In this section, we apply the proposed methods to test functions. Five test functions in different conditions were studied for a comparative analysis of the methods. These test function are actually “toy models”, that is, simple functions which are in fact easy to compute. We however treat these functions as if they are computationally expensive real simulators. Those functions are introduced only to evaluate and to compare the performances of the proposed methods. Test functions 1 and 2 are simple with one or two dimensional problems, while test functions 4 and 5 involve three or four dimensional problems. A real simulator which takes long time to compute is considered in the next section.

The experimental data with $n_{E}$ sample size and the computer experiment with $n_{S}$ sample size were generated by

$$y_{E}=Y\left(c^{*},x_{E}\right)+e,$$

and

$$y_{S}=Y\left(c_{S},x_{S}\right).$$

The five test functions along with the true values of parameters c are as follows:

**Test function 1**: $Y\left(c,x\right)=c_{1}x_{1}exp\left(-c_{1}x_{2}\right)$,
  $\;c^{*}=\left(2\right),\;n_{E}=20,\;e∼N\left(0,0.05^{2}\right)$, $n_{S}=20$
**Test function 2**: $Y\left(c,x\right)=c_{1}x_{1}+c_{2}x_{2}^{2}$,
  $\;c^{*}=\left(1,1\right),\;n_{E}=30,\;e∼N\left(0,0.05^{2}\right)$, $n_{S}=30$
**Test function 3**: $Y\left(c,x\right)=c_{1}x_{1}+sin\left(c_{2}+x_{2}\right)+c_{2}x_{2}^{2}$,
  $\;c^{*}=\left(2,1\right),\;n_{E}=50,\;e∼N\left(0,\sigma_{E}^{2}\right),$
  $\;\sigma_{E}^{2}=0.01{\left(y_{E}-5\right)}^{2}+0.01,\;n_{S}=50$
**Test function 4**: $Y\left(c,x\right)=c_{1}x_{1}+c_{2}x_{2}^{2}+c_{3}x_{3}^{3}$,
  $\;c^{*}=\left(3,1,2\right),\;n_{E}=80,\;e∼N\left(0,\sigma_{E}^{2}\right),$,
  $\;\sigma_{E}^{2}=0.005\left|y_{E}\right|+0.005,\;n_{S}=80$
**Test function 5**: $Y\left(c,x\right)=c_{1}x_{1}^{2}+c_{2}x_{2}+c_{3}cos\left(x_{3}\pi \right)+c_{4}sin\left(x_{4}\pi \right)$,
  $\;c^{*}=\left(1,1,3,3\right),\;n_{E}=150,\;e∼N\left(0,\sigma_{E}^{2}\right),$,
  $\;\sigma_{E}^{2}=0.00005{\left(y_{E}-5\right)}^{2}{\left(y_{E}-30\right)}^{2}+0.005,\;n_{S}=150$

Table 1 shows the result of the estimation by the nonlinear least squares (NLS) method with the true test functions. The last column shows how many numbers of evaluations are needed to estimate based on the NLS using simulation code directly. As the simulation code becomes more complex, the number of evaluations required increases dramatically. So, if the simulation code very time consuming, the estimation based on the NLS is impossible in terms of computation time. With the five test functions, we examined how well the four methods estimate the parameters in ‘fast’ simulation codes using only a small number of simulated data.

The optimal Latin-hypercube designs [31,32] were used to select an independent variable for real experiment ($x_{E}$) and an input variable for the computer simulations $\left(c_{S},x_{S}\right)$. To address uncertainty in the design, 30 different computer experimental designs were used for each test function, while the real experimental design was fixed. If this design were not fixed, the variability of *c* estimation would increase.

For a comparison analysis, we provide the average of 30 different estimates and the standard deviation of the calibrated parameter values. The averaged distance to the true values from the estimates is calculated to evaluate the accuracy of the methods. To take account of both the variance and the accuracy of estimates, we also considered the root mean squared error (RMSE) of the estimates as a performance measure. The formula of RMSE is as follows:

(15) $$RMSE\left(\hat{c}\right)=\sqrt{Bias^{2}\left(\hat{c}\right)+\sum_{i=1}^{q}\left(std({\hat{c}}_{i}\right){)}^{2}},$$

where $Bias(\hat{c})$ is the averaged distance to true values and $std({\hat{c}}_{i})$ is the standard deviation of each estimates obtained from 30 repetitions. In addition, the computing time is also provided with the unit of seconds. The test function study was conducted using a PC on an Intel i5 CPU (3.6 GHz) with 16 GB of memory.

### Iteratively Re-Weighted ALS

When the errors do not have equal error variance in the regression analysis, one can use the weighted least squares method. The weights can be determined by an iteratively, which leads to the iteratively re-weigthed least squares method (IRLS). It has been employed for robust regression for obtaining estimated regression coefficients that are relatively unaffected by extreme observations. In the IRLS, the weights are functions of the residuals from the previous iteration such that points with larger residuals receive relatively less weight than points with smaller residuals. Consequently unusual points tend to receive less weight than typical points [33].

We now apply IRLS method to the ALS setting for calibration of computer code, which is called as the iteratively re-weighted ALS (IRWALS). The algorithm of the IRWALS is as follows:

**Step 1**: Get ${\hat{c}}^{\left(0\right)}$ using the ALS, which minimizes the non-weighted residual sum of squares
  (16) $$ARSS\left(c\right)=\sum_{i=1}^{n_{E}}{\left[y_{E,i}-\hat{Y}\left(c,x_{E,i}\right)\right]}^{2}.$$
**Step 2**: Using the previously estimated parameters ${\hat{c}}^{\left(t-1\right)}$, calculate the *t*-th weight
  (17) $$w_{i}^{\left(t\right)}={\left|y_{E,i}-\hat{Y}\left({\hat{c}}^{\left(t-1\right)},x_{E,i}\right)\right|}^{p-2},$$
  where $i=1,...,n_{E}$. In this study, we consider the case p=1.
**Step 3**: Calculate t-th estimates ${\hat{c}}^{\left(t\right)}$ using the weighted ARSS
  (18) $$WARSS\left(c\right)={\left[y_{E}-\hat{Y}\left(T_{E}\right)\right]}^{\prime }W^{\left(t\right)}\left[y_{E}-\hat{Y}\left(T_{E}\right)\right],$$
  where $W^{\left(t\right)}$ is the diagonal matrix with entries $w_{1}^{\left(t\right)},...,w_{n_{E}}^{\left(t\right)}$.
**Step 4**: Repeat Steps 2 and 3 until $\sum_{i=1}^{n_{E}}{\left(w_{i}^{\left(t\right)}-w_{i}^{\left(t-1\right)}\right)}^{2}/\sum_{i=1}^{n_{E}}{\left(w_{i}^{\left(t\right)}\right)}^{2}<k$.

The IRWALS method is similar to the case in which the matrix K in the GALS is a diagonal. Thus, it is expected that the IRWALS may perform better than the ALS when errors do not follow equal variances. Note that, however, the IRWALS method still has the same drawback as in the ALS that it uses the surrogate $\hat{Y}$ which was built only once from the computer data and is not updated thereafter. Whereas the max-min or the max-minG algorithms update the surrogate $\hat{Y}$ iteratively using both computer data and experimental data. The IRWALS method has this difference with the max-min or the max-minG algorithms. It thus may not share the advantage of the max-min type algorithm.

Table 2, Table 3, Table 4, Table 5 and Table 6 show the results of the performance comparison for each test function. In terms of the RMSE, the proposed methods (the GALS and the max-minG) offer better results than the ALS and IRWALS, overall. Especially, the max-minG offers more accurate results (in terms of the averaged distance to true values) and less RMSE than the others including GALS in relatively complex cases such as test functions 4 and 5.

**Figure 2:**
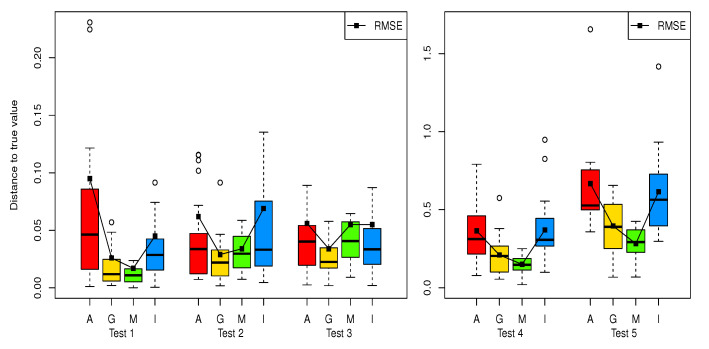
Boxplot of distance to true value in five test functions. The acronyms A, G, M, and I at the bottom stand for the ALS, GALS, max-minG and IRWALS methods, respectively.

**Figure A1:**
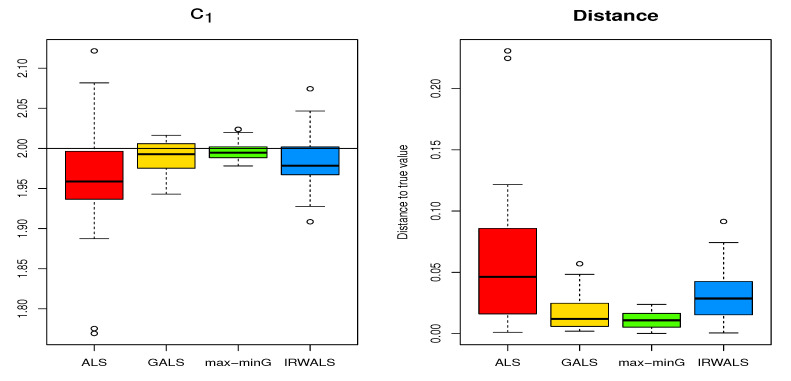
Boxplot of estimated values and a boxplot of the distances between true and estimated values for test function 1.

**Figure A2:**
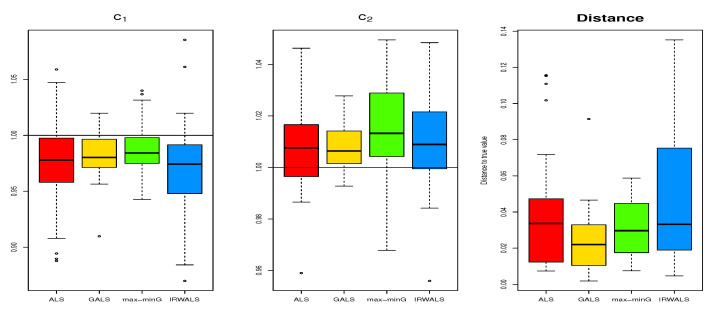
Same as Figure A1 but for test function 2.

**Figure A3:**
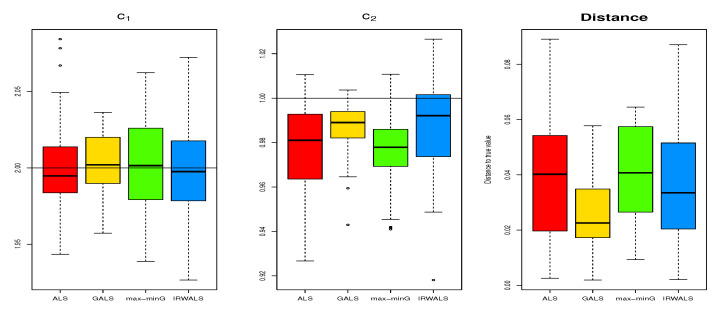
Same as Figure A1 but for test function 3.

**Figure A4:**
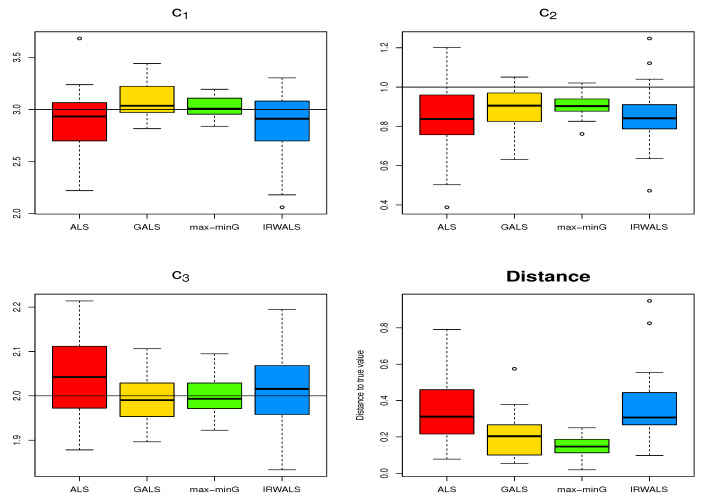
Same as Figure A1 but for test function 4.

**Figure A5:**
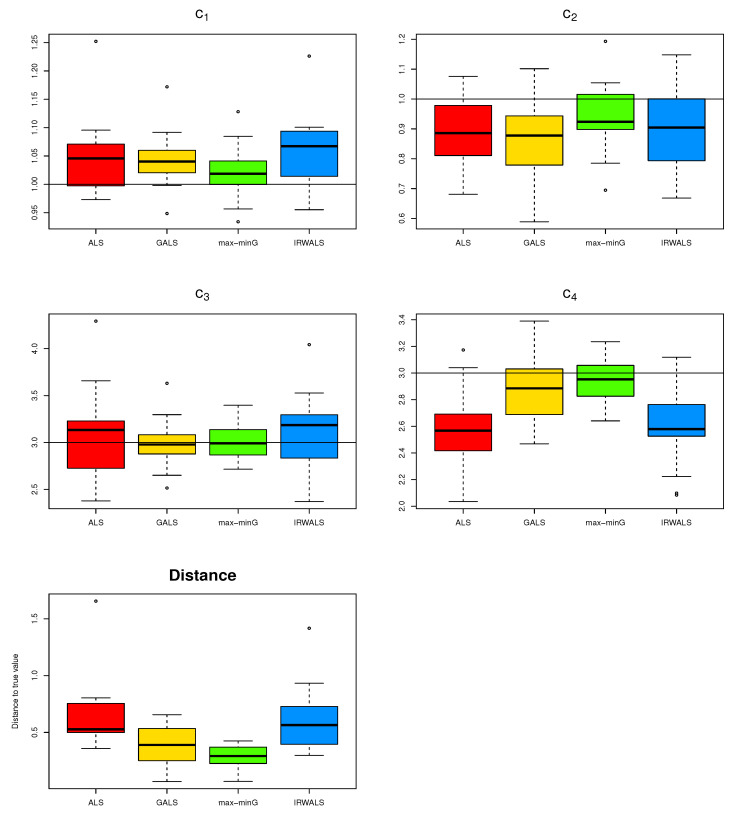
Same as Figure A1 but for test function 5.

This is also confirmed in Figure 2 which shows the boxplot of distance to true value with the RMSE for the five test functions. The details are shown in Figure A1, Figure A2, Figure A3, Figure A4 and Figure A5, where the last boxplots show the distances between the estimates and true values, and the other boxplots show estimates with true values (horizontal solid line). In most cases, the medians of the estimates obtained from the GALS and max-minG are closer to the true values than those from the ALS and IRWALS. Furthermore, the box lengths of the estimates obtained from the GALS and max-minG are shorter than those from the ALS and the IRWALS. From the boxplot of the distances between the estimates and true values, it is also confirmed that the GALS and max-minG give more accurate and stable (shorter box lengths) results in overall.

**Figure 3:**
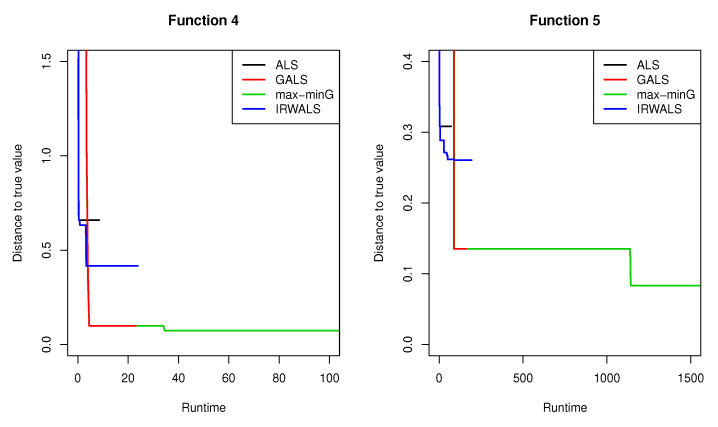
The performance plots over runtime in terms of distances to true values for test functions 4 and 5 (unit: second).

The computing time of the max-minG, however, is much longer than the other methods (see Table 2, Table 3, Table 4, Table 5 and Table 6). Figure 3 shows how the performance of each model changes over the runtime (unit: second) on sample cases for test functions 4 and 5. The plots were truncated at run time of 100 and 1500 s to see the beginning part in detail.

## 7. Application to a Nuclear Fusion Simulator

In this section, we apply the proposed methods to a complex computer code called Baldur [34] which is a computationally expensive simulator of energy confinement time in a nuclear fusion device (called a tokamak from the Russian language). The model can be written as follows:

(19) $$y=f(c_{1},c_{2},c_{3},c_{4},x_{1},x_{2},x_{3},x_{4}),$$

where *f* is a known function calculated by the Baldur code, $x_{1}$ is the input heating power, $x_{2}$ is the toroidal plasma current, $x_{3}$ is the line average electron density, $x_{4}$ is the toroidal magnetic field, and $c=(c_{1},c_{2},c_{3},c_{4})$ are adjustable parameters determining energy transfer: drift waves, rippling, resistive ballooning, and the critical value of $\eta_{i}$ (which provokes increased ion energy losses for the drift), respectively.

The experimental data consist of only $x=\{x_{1},x_{2},x_{3},x_{4}\}$, whereas the computer data consist of (c,x). We use 42 observations from PDX (Poloidal Divertor Experiment) tokamak in Princeton and 64 responses from the Baldur simulator. A detailed description of the data is found in References [3,34]. We use a simple model as follows:

(20) $$Y\left(x\right)=\beta_{0}+\beta_{1}t_{1}+...+\beta_{d}t_{d}+Z\left(t\right)+\varepsilon ,\mathrm{with}\;a\;\mathrm{common}\theta ,$$

where a common θ is that $\theta_{1}=...=\theta_{d}=\theta$.

Table 7 shows the estimates of the tuning parameters and their 90% confidence intervals within the parentheses obtained by various tuning methods from the tokamak data. The confidence interval was calculated by the parametric bootstrap with B = 1000.

**Figure 4:**
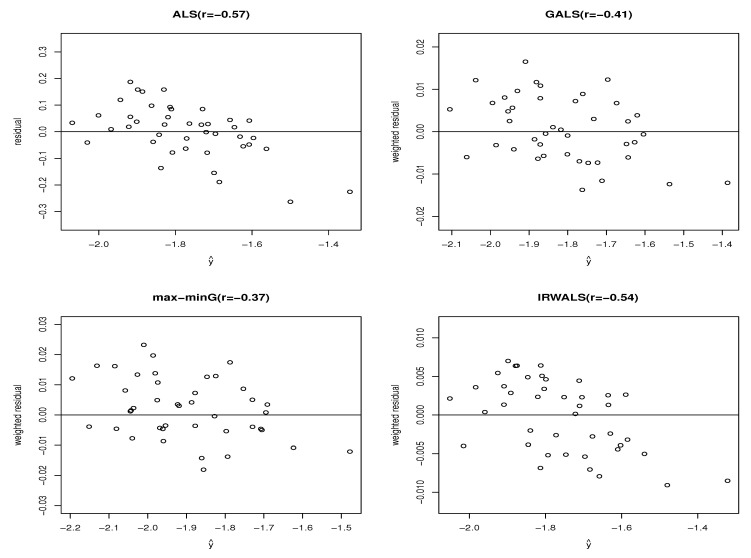
The residuals resulted from the ALS, and the weighted residuals from the GALS, the max-minG algorithm, and the IRWALS for the nuclear fusion experiments data. The values within the parentheses are the Pearson correlation coefficients (r) between the residuals (or weighted residuals) and the predicted values obtained from each method.

Figure 4 shows the residuals ($y_{E}-\hat{Y}\left(T_{E}\right)$), weighted residuals (${\hat{K}}^{-1/2}\left(y_{E}-\hat{Y}\left(T_{E}\right)\right)$, and ${\hat{W}}^{-1/2}\left(y_{E}-\hat{Y}\left(T_{E}\right)\right)$) according to the predited values $\hat{Y}\left(T_{E}\right)$. The residuals from the ALS show a pattern of declining as $\hat{Y}\left(T_{E}\right)$ increases. Whereas the abnormal pattern in the weighted residuals from the GALS and the max-minG is hardly observed. It can be confirmed via the Pearson correlation coefficient between the residuals (or the weighted residuals) and the predicted values from each method in Figure 4. The correlation coefficient, which was −0.57 on the ALS, barely changed to −0.54 on the IRWASL, but significantly changed to −0.41 and −0.37 on the GALS and max-minG, respectively.

## 8. Discussion

Unlike traditional estimations in statistics, we have two kinds of data (real data and computer data) for calibration. Thus, the following issues were considered for the calculation of $\hat{K}$. First, $R(T_{E},T_{E})$, and $\gamma_{E}^{2}$ are related to a real experiment, and $V_{S}$ is related to the computer data. In addition, *U* and $\sigma_{Z}^{2}$ are related to both data sets. In this sense, it seems reasonable to estimate $R\left(T_{E},T_{E}\right),\gamma_{E}^{2}$, and $V_{S}$ using the related data set, and to estimate *U* and $\sigma_{Z}^{2}$ using the combined data. However, we have the tuning parameter $c_{S}$ for the computer simulations, but we do not have $c_{E}$ for the real data; hence, it is not easy to estimate $U,\sigma_{Z}^{2}$ using the combined data. From a simple test, it was confirmed that ${\hat{\theta }}^{\prime }$ estimated using the real data can be vastly different from those using computer data; thus, it may not lead to good calibration results. Therefore, we used $\hat{\theta }$ estimated from computer data for $R(T_{E},T_{E}),U,$ and $V_{S}$, while we used ${\hat{\gamma }}_{E}$ estimated from real data. We do not claim that this is the final answer. One may try another version of ${\hat{\gamma }}_{E}$ in constructing $\hat{K}$. For example, leave-one-out or *k*-fold cross validations would be useful to select an appropriate $\gamma_{E}$ in *K*.

There are basic limitations with tuning computer code to real-world data regarding the experimental design. We found that the performance of tuning methods is significantly dependent on the designs for both the computer experiments and the physical experiments. Some authors including [35,36] explored this topic. We agree that a sequential tuning approach is practically useful, as in References [13,15,20]. Further research on relevant designs under sequential tuning approach will be helpful.

The estimates $\hat{c}$ may vary according to the selected metamodel; hence, the selection of the model is very important in calibration. In this study, we only use the simple model (Equation (7)) for the simulation study. If the best model selected by the variable selection method was used, the result might be better. In this study, we did not consider the identifiability of the tuning parameter, which is an important focus of recent developments [16,17].

In this study, we assume the computer code is deterministic so that the responses will not be changed for the same input. If the code is stochastic, our results may be changed. In the latter case, the uncertainty of *c* estimation would increase and need more sample sizes to have similar precision as we have in this study.

One issue in the GP modeling involves manupulations of an n×n covariance matrix that require $O(n^{3})$ computations, where *n* is the sample size. The calculation is computationally intensive and often intractable in big data. Various computationally efficient approximations have been proposed to address this problem. The sparse approximations employ m(m≪n) inducing points to summarize the whole training data. It reduces the complexity to $O(nm^{2})$. Another method is the aggregation of models, also known as consensus statistical method. This kind of scheme produces the final predictions by the aggregation of *M* submodels respectively trained on the random subsets or the disjoint partitions of data, thus distributing the computations to the experts [37] who still cover the whole space with moderate sample size. The third method is a local approximate GP which searches for the most useful training data points – a subdesign relative *x* such as nearest neighbor subset – for predicting at *x*, without considering/handling large matrices [28]. For big data, the calibration algorithms proposed in this study can be useful with modification to the above computational approximations. Parallelization in implementing a calibration algorithm for big data is essential to taking full advantage of contemporary computer architectures.

## 9. Summary

The approximated nonlinear least squares method (ALS) using a Gaussian surrogate is an ordinary calibration technique for complex computer code. It however has potential drawbacks of not dealing with the correlation in the residuals and of not taking into account the uncertainty due to using a surrogate to computer code. To address these difficulties, we proposed a generalized approximated nonlinear least squares method (GALS) for tuning complex computer codes. We also introduced the iterative algorithm of the GALS which is named as the max-minG. In addition, the iteratively re-weighted ALS method (IRWALS) was considered for a comparison purpose.

For the performance comparison, we examined the five test functions including the cases in which real experiment data has unequal variance error. From the results of the test function study, we confirmed that our proposed methods (the GALS and the max-minG) provide better results than the ALS and the IRWALS in terms of the root mean squared error (RMSE). In particular, in the test functions 4 and 5, which are relatively complex and unequal variance cases, the max-minG provided more accurate estimates and the variance of the estimates are smaller than those of the others including the GALS. In addition, we also identified that the abnormal pattern of residuals in the ALS can be resolved by using the proposed methods in an application to a nuclear fusion simulator.

The disadvantage of the proposed methods (the GALS and the max-minG) is that those are computationally more complex than the ALS, because the GALS and the max-minG need to optimize more complex functions and needs to estimate more parameters for the covariance matrix *K*. Nonetheless, the GALS and the max-minG may provide a better code tuing as well as a better surrogate of the computer code than the ALS or the IRWALS method can do.

## Figures and Tables

**Table 1:** Estimates and the distances to true values for the test functions 1–5 by using the nonlinear least squares with the true functions. The values within the parentheses are the true values for each parameter. The last column is the number of simulation code evaluation for the estimation of each function with a single initial value.

Function	${\hat{c}}_{1}\left(c_{1}\right)$	${\hat{c}}_{2}\left(c_{2}\right)$	${\hat{c}}_{3}\left(c_{3}\right)$	${\hat{c}}_{4}\left(c_{4}\right)$	Distance to True Value	# of Evaluation
Test function 1	2.002(2)				0.002	360
Test function 2	0.992 (1)	1.003 (1)			0.008	1200
Test function 3	1.987 (2)	0.986 (1)			0.019	2000
Test function 4	2.983 (3)	0.997 (1)	2.004 (1)		0.018	5040
Test function 5	1.002 (1)	0.987 (1)	2.994 (3)	2.997 (3)	0.014	27,000

**Table 2:** Result for the test function 1 with the true values $c^{*}=\left(2\right)$. The values are the averaged estimates of $c^{*}$, the averaged distance to the true values from the estimates, the root mean squared error (RMSE) of the estimates, and the computing time of doing the calibration with the unit of seconds. The numbers in parentheses are the standard deviations.

Method	Mean of ${\hat{c}}_{1}$	Mean Distance to True Value	RMSE	Mean Time of Calibration
ALS	1.959 (0.073)	0.061 (0.057)	0.095	0.70
GALS	1.989 (0.020)	0.017 (0.015)	0.026	1.2
max-minG	1.997 (0.013)	0.011 (0.007)	0.017	7.4
IRWALS	1.985 (0.034)	0.030 (0.022)	0.045	1.0

**Table 3:** Same as Figure 2 but for test function 2 with $c^{*}=\left(1,1\right)$.

Method	Mean of ${\hat{c}}_{1}$	Mean of ${\hat{c}}_{2}$	Mean Distance to True Value	RMSE	Mean Time of Calibration
ALS	0.976 (0.043)	1.008 (0.018)	0.041 (0.033)	0.062	1.6
GALS	0.981 (0.022)	1.008 (0.009)	0.024 (0.018)	0.029	5.1
max-minG	0.988 (0.024)	1.016 (0.018)	0.032 (0.016)	0.034	20.4
IRWALS	0.972 (0.047)	1.010 (0.02)	0.047 (0.036)	0.069	2.8

**Table 4:** Same as Figure 2 but for test function 3 with $c^{*}=\left(2,1\right)$.

Method	Mean of ${\hat{c}}_{1}$	Mean of ${\hat{c}}_{2}$	Mean Distance to True Value	RMSE	Mean Time of Calibration
ALS	2.001 (0.035)	0.976 (0.02)	0.040 (0.024)	0.056	2.1
GALS	2.004 (0.02)	0.985 (0.014)	0.025 (0.013)	0.034	9.8
max-minG	2.001 (0.033)	0.976 (0.018)	0.041 (0.017)	0.055	72.5
IRWALS	1.997 (0.034)	0.987 (0.023)	0.037 (0.022)	0.055	4.2

**Table 5:** Same as Figure 2 but for test function 4 with $c^{*}=\left(3,1,2\right)$.

Method	Mean of ${\hat{c}}_{1}$	Mean of ${\hat{c}}_{2}$	Mean of ${\hat{c}}_{3}$	Mean Distance to True Value	RMSE	Mean Time of Calibration
ALS	2.894 (0.316)	0.831 (0.183)	2.031 (0.09)	0.365 (0.212)	0.524	8.9
GALS	3.083 (0.164)	0.889 (0.109)	2.002 (0.018)	0.210 (0.125)	0.198	39.3
max-minG	3.023 (0.108)	0.909 (0.062)	1.997 (0.044)	0.150 (0.055)	0.132	183.2
IRWALS	2.869 (0.335)	0.861 (0.165)	2.010 (0.086)	0.370 (0.204)	0.383	23.7

**Table 6:** Same as Figure 2 but for test function 5 with $c^{*}=\left(1,1,3,3\right)$.

Method	Mean of ${\hat{c}}_{1}$	Mean of ${\hat{c}}_{2}$	Mean of ${\hat{c}}_{3}$	Mean of ${\hat{c}}_{4}$	Mean Distance to True Value	RMSE	Mean Time of Calibration
ALS	1.050 (0.069)	0.886 (0.121)	3.081 (0.494)	2.572 (0.298)	0.667 (0.311)	0.892	47
GALS	1.044 (0.049)	0.867 (0.133)	2.998 (0.269)	2.895 (0.267)	0.396 (0.17)	0.304	149
max-minG	1.023 (0.048)	0.941 (0.122)	3.011 (0.2)	2.949 (0.176)	0.282 (0.102)	0.239	3263
IRWALS	1.058 (0.067)	0.895 (0.141)	3.107 (0.427)	2.611 (0.307)	0.615 (0.292)	0.454	123

**Table 7:** Estimates of the tuning parameters and their 90% confidence intervals within the parentheses obtained by various tuning methods from the nuclear fusion experiments data. The last column shows the computing time of doing the calibration with the unit of seconds.

Method	${\hat{c}}_{1}$	${\hat{c}}_{2}$	${\hat{c}}_{3}$	${\hat{c}}_{4}$	Time of Calibration
ALS	1.213 (1.029, 1.670)	3.092 (1.284, 4.088)	1.050 (0.308, 1.446)	0.342 (0.005, 1.411)	16
GALS	1.474 (0.901, 1.860)	3.997 (1.612, 4.698)	0.899 (0.353, 1.622)	0.438 (0.000, 1.966)	68
max-minG	1.172 (0.454, 1.186)	4.653 (0.554, 4.933)	0.942 (0.337, 1.943)	0.755 (0.000, 1.995)	320
IRWALS	1.064 (0.889, 1.811)	3.220 (0.842, 4.054)	1.088 (0.125, 1.562)	0.296 (0.000, 1.888)	57

## Appendix A. Calculation of the Covariance Matrix K

In this section, we calculate the $n_{E}\times n_{E}$ covariance matrix of residual $y_{E}-\hat{Y}\left(c,x_{E}\right)$, which is given by

(A1) $$K=E\left[\left(y_{E}-\hat{Y}\left(T_{E}\right)\right){\left(y_{E}-\hat{Y}\left(T_{E}\right)\right)}^{\prime }\right],$$

where $n_{E}\times d$ matrix $T_{E}={\left\{t_{E,1},t_{E,2},...,t_{E,n_{E}}\right\}}^{\prime }$ for $t_{E,i}^{\prime }=\left(c,x_{E,i}\right),i=1,...,n_{E}$. Since $y_{E}-\hat{Y}\left(T_{E}\right)=y_{E}-Y\left(T_{E}\right)+Y\left(T_{E}\right)-\hat{Y}\left(T_{E}\right)$, K is partitioned into four pieces:

(A2) $$\begin{array}{rr} K= & E\left[\left(y_{E}-Y\left(T_{E}\right)\right){\left(y_{E}-Y\left(T_{E}\right)\right)}^{\prime }\right]+E\left[\left(y_{E}-Y\left(T_{E}\right)\right){\left(Y\left(T_{E}\right)-\hat{Y}\left(T_{E}\right)\right)}^{\prime }\right]+\\ & E\left[\left(Y\left(T_{E}\right)-\hat{Y}\left(T_{E}\right)\right){\left(y_{E}-Y\left(T_{E}\right)\right)}^{\prime }\right]+E\left[\left(Y\left(T_{E}\right)-\hat{Y}\left(T_{E}\right)\right){\left(Y\left(T_{E}\right)-\hat{Y}\left(T_{E}\right)\right)}^{\prime }\right]. \end{array}$$

We assume that $e=y_{E}-Y\left(T_{E}\right)$ is independent of $Y\left(T_{E}\right),\hat{Y}\left(T_{E}\right)$, and $y_{E}$. Assumming that E(e)=0, we have

(A3) $$E\left[\left(y_{E}-Y\left(T_{E}\right)\right){\left(Y\left(T_{E}\right)-\hat{Y}\left(T_{E}\right)\right)}^{\prime }\right]=E\left[e\cdot Y\left(T_{E}\right)\right]-E\left[e\cdot \hat{Y}\left(T_{E}\right)\right]=0.$$

Therefore, *K* is given by

(A4) $$K=\sigma_{E}^{2}I+\Sigma \left(T_{E}\right),$$

where $\Sigma \left(T_{E}\right)=E\left[\left(Y\left(T_{E}\right)-\hat{Y}\left(T_{E}\right)\right){\left(Y\left(T_{E}\right)-\hat{Y}\left(T_{E}\right)\right)}^{\prime }\right]$, a $n_{E}\times n_{E}$ matrix. For the calculation of $\Sigma (T_{E})$, observe that from Equation (6)

(A5) $$\hat{Y}\left(T_{E}\right)=\left(U,F_{E}\right){\left[\begin{array}{cc} V_{S} & F_{S}\\ F_{S}^{\prime } & 0 \end{array}\right]}^{-1}{\left(\begin{array}{c} y_{S}^{\prime }\\ \;0 \end{array}\right)}^{\prime },$$

using the same notations denoted in the Section 4.

We decompose $\Sigma (T_{E})$ into the following;

(A6) $$\begin{array}{rrr} \Sigma (T_{E}) & = & E[\left(Y\left(T_{E}\right)-\hat{Y}\left(T_{E}\right)\right){\left(Y\left(T_{E}\right)-\hat{Y}\left(T_{E}\right)\right)}^{\prime }]\\ & = & E[\left(Y\left(T_{E}\right)Y^{\prime }\left(T_{E}\right)\right]-E[\left(Y\left(T_{E}\right){\hat{Y}}^{\prime }\left(T_{E}\right)\right]\\ & & \;-E[\left(\hat{Y}\left(T_{E}\right)Y^{\prime }\left(T_{E}\right)\right]+E[\left(\hat{Y}\left(T_{E}\right){\hat{Y}}^{\prime }\left(T_{E}\right)\right]. \end{array}$$

For the first term of Equation (A6), since $Y\left(T_{E}\right)=F_{E}\beta +Z\left(T_{E}\right)$ and $E[Z\left(T_{E}\right)]=0$, we obtain

(A7) $$E[\left(Y\left(T_{E}\right)Y^{\prime }\left(T_{E}\right)\right]=E\left[\left(F_{E}\beta +Z\left(T_{E}\right)\right){\left(F_{E}\beta +Z\left(T_{E}\right)\right)}^{\prime }\right]=F_{E}\beta \beta^{\prime }F_{E}^{\prime }+\sigma_{Z}^{2}R\left(T_{E},T_{E}\right).$$

Using Equation (A5) and the inverse formula for a block matrix [38], the third and fourth terms of Equation (A6) are given by

(A8) $$E[\left(\hat{Y}\left(T_{E}\right)Y^{\prime }\left(T_{E}\right)\right]=\sigma_{Z}^{2}UV_{S}^{-1}\left(I-F_{S}GF_{S}^{\prime }V_{S}^{-1}\right)U^{\prime }+F_{E}\beta \beta^{\prime }F_{E}^{\prime }+\sigma_{Z}^{2}F_{E}GF_{S}^{\prime }V_{S}^{-1}U^{\prime }$$

and

(A9) $$E[\left(\hat{Y}\left(T_{E}\right){\hat{Y}}^{\prime }\left(T_{E}\right)\right]=\sigma_{Z}^{2}UV_{S}^{-1}\left(I-F_{S}GF_{S}^{\prime }V_{S}^{-1}\right)U^{\prime }+F_{E}\left(\beta \beta^{\prime }F_{E}+\sigma_{Z}^{2}G\right)F_{E}^{\prime },$$

where $G={\left(F_{S}V_{S}^{-1}F_{S}\right)}^{-1}$. From Equations (A7)–(A9), we have

(A10) $$\begin{array}{rrr} \Sigma (T_{E}) & = & \sigma_{Z}^{2}(R\left(T_{E},T_{E}\right)-UV_{S}^{-1}U^{\prime }+UV_{S}^{-1}F_{S}GF_{S}^{\prime }V_{S}^{-1}U^{\prime }\\ & & -F_{E}GF_{S}^{\prime }V_{S}^{-1}U^{\prime }-UV_{S}^{-1}F_{S}G^{\prime }F_{E}^{\prime }+F_{E}GF_{E}^{\prime }), \end{array}$$

and it is rearranged as the following by the inverse formula for a block matrix again;

(A11) $$\sigma_{Z}^{-2}\Sigma \left(T_{E}\right)=R\left(T_{E},T_{E}\right)-\left(U,F_{E}\right){\left[\begin{array}{cc} V_{S} & F_{S}\\ F_{S}^{\prime } & 0 \end{array}\right]}^{-1}\left(\begin{array}{c} U^{\prime }\\ \;F_{E}^{\prime } \end{array}\right).$$

Finally, K is written as, for $\gamma_{E}=\sigma_{E}^{2}/\sigma_{Z}^{2}$

(A12) $$K=\sigma_{Z}^{2}\left(\sigma_{Z}^{-2}\Sigma \left(T_{E}\right)+\gamma_{E}I\right),$$

or

(A13) $$K=\sigma_{E}^{2}I+\Sigma \left(T_{E}\right).$$
